# Drug-Induced Liver Injury Secondary to Meropenem: Diagnostic Challenge and Therapeutic Role of Hemoadsorption

**DOI:** 10.7759/cureus.96206

**Published:** 2025-11-06

**Authors:** Leonor Simões, Miguel Sequeira, Carlos Silva, Catarina Monteiro

**Affiliations:** 1 Intensive Care Unit, Centro Hospitalar e Universitário de Coimbra - Unidade Local de Saúde de Coimbra, Coimbra, PRT; 2 Internal Medicine, Centro Hospitalar e Universitário de Coimbra - Unidade Local de Saúde de Coimbra, Coimbra, PRT

**Keywords:** cholestatic liver injury, critical care, drug induced liver injury (dili), hemoadsorption, intensive care, meropenem therapy

## Abstract

Meropenem is a broad-spectrum carbapenem antibiotic commonly used in critically ill patients. While generally considered safe, rare cases of drug-induced liver injury (DILI) have been reported. We describe a case of intrahepatic cholestatic hepatitis attributed to meropenem in an intensive care unit (ICU) patient, with histologic confirmation and clinical improvement following hemoadsorption therapy. A 65-year-old man was admitted to the ICU following a fall from height, sustaining multiple traumatic injuries, including spinal cord trauma with neurogenic shock. He developed pneumonia requiring escalation to meropenem. Five days into therapy, progressive cholestasis and hyperbilirubinemia developed, despite no biliary obstruction on imaging. Meropenem was discontinued on day 12 of treatment. Liver function continued to deteriorate, culminating in hepatic encephalopathy. Hemoadsorption therapy (HA-380 cartridge) was initiated with clinical and biochemical improvement. Liver biopsy confirmed intrahepatic cholestatic hepatitis consistent with DILI. After recurrence of symptoms and worsening cholestasis, hemoadsorption was restarted with resolution of pruritus and improved liver function. Meropenem-induced liver injury is rare and usually self-limiting. In our patient, liver injury followed a cholestatic pattern, confirmed histologically, and likely represented an idiosyncratic reaction. The recurrence of liver dysfunction upon cessation of hemoadsorption and subsequent response to therapy suggests a possible therapeutic role of hemoadsorption in managing hyperbilirubinemia and cholestatic symptoms in DILI. This case highlights a rare but severe presentation of meropenem-induced cholestatic hepatitis in a critically ill patient. Hemoadsorption may be a valuable adjunctive therapy in select cases of DILI with significant bilirubin elevation and pruritus. Clinicians should maintain a high index of suspicion for DILI in ICU patients receiving broad-spectrum antibiotics.

## Introduction

Meropenem is a broad-spectrum carbapenem antibiotic widely used in the hospital setting for the treatment of severe infections, including those involving multidrug-resistant organisms. It exhibits strong activity against a range of gram-positive, gram-negative, and anaerobic bacteria. Despite its extensive use and general safety profile, meropenem has been associated with mild, transient elevations in aminotransferases, and in rare instances, with clinically apparent drug-induced liver injury (DILI), particularly of a cholestatic pattern [1].

DILI is a diagnosis of exclusion and a significant cause of morbidity [2,3]. Its idiosyncratic form (iDILI) presents a diagnostic challenge due to its unpredictable latency and variable clinical presentation [4]. The few published cases of DILI related to meropenem describe cholestatic liver injury manifesting within a few days to three weeks of therapy, and in very rare cases, progression to vanishing bile duct syndrome has been reported [5-7].

To date, no case has been described linking meropenem-induced liver injury with the concurrent use of hemoadsorption therapy. Hemoadsorption, particularly using the HA-380 cartridge, is an emerging extracorporeal treatment aimed at removing inflammatory mediators, toxins, and protein-bound solutes from the circulation [8]. While primarily employed in septic patients with vasoplegia, its applications have expanded to include management of acute liver failure, intractable cholestatic pruritus, and drug intoxications [9,10]. The case we report is, to our knowledge, the first to describe severe cholestatic liver injury potentially triggered by meropenem, with clinical improvement following hemoadsorption therapy.

## Case presentation

A 65-year-old previously independent male with a medical history of hypertension, heart failure with reduced ejection fraction, atrial fibrillation on anticoagulation, and dyslipidemia, was admitted to the intensive care unit (ICU) following a work-related fall from a height of three meters.

On presentation, he sustained a mild traumatic brain injury with an acute subdural hematoma, facial trauma with periorbital and mid-facial fractures, thoracic trauma with bilateral rib fractures, pulmonary contusions, a left-sided hemothorax requiring drainage, and a traumatic vertebral fracture at D4, resulting in neurogenic shock and complete paraplegia (American Spinal Injury Association (ASIA) A).

During the early ICU course, the patient developed pneumonia, presumed to be community-acquired, and was started empirically on amoxicillin-clavulanic acid. Due to clinical and radiological deterioration without microbiological identification, antimicrobial therapy was escalated to meropenem and vancomycin on day 7 of admission. Subsequent cultures from tracheobronchial aspirate identified *Acinetobacter baumannii* and extended-spectrum beta-lactamase (ESBL)-producing *Escherichia coli*. Vancomycin was discontinued, and meropenem was continued at a dose of 1 g every 12 hours.

The patient showed progressive clinical improvement in respiratory and infectious parameters. However, from day 11 of admission (day 5 of meropenem therapy), laboratory evaluation revealed increasing cholestatic liver enzymes and hyperbilirubinemia (R factor = 0.5). After reviewing the patient’s medication, the Roussel Uclaf Causality Assessment Method (RUCAM) scale considers DILI due to meropenem as possible (five points).

An abdominal ultrasound performed on day 12 showed hepatomegaly (22 cm in longitudinal axis), with regular contours and homogeneous texture, though increased parenchymal echogenicity suggestive of metabolic overload. No biliary dilation or other abnormalities were noted.

**Figure 1:**
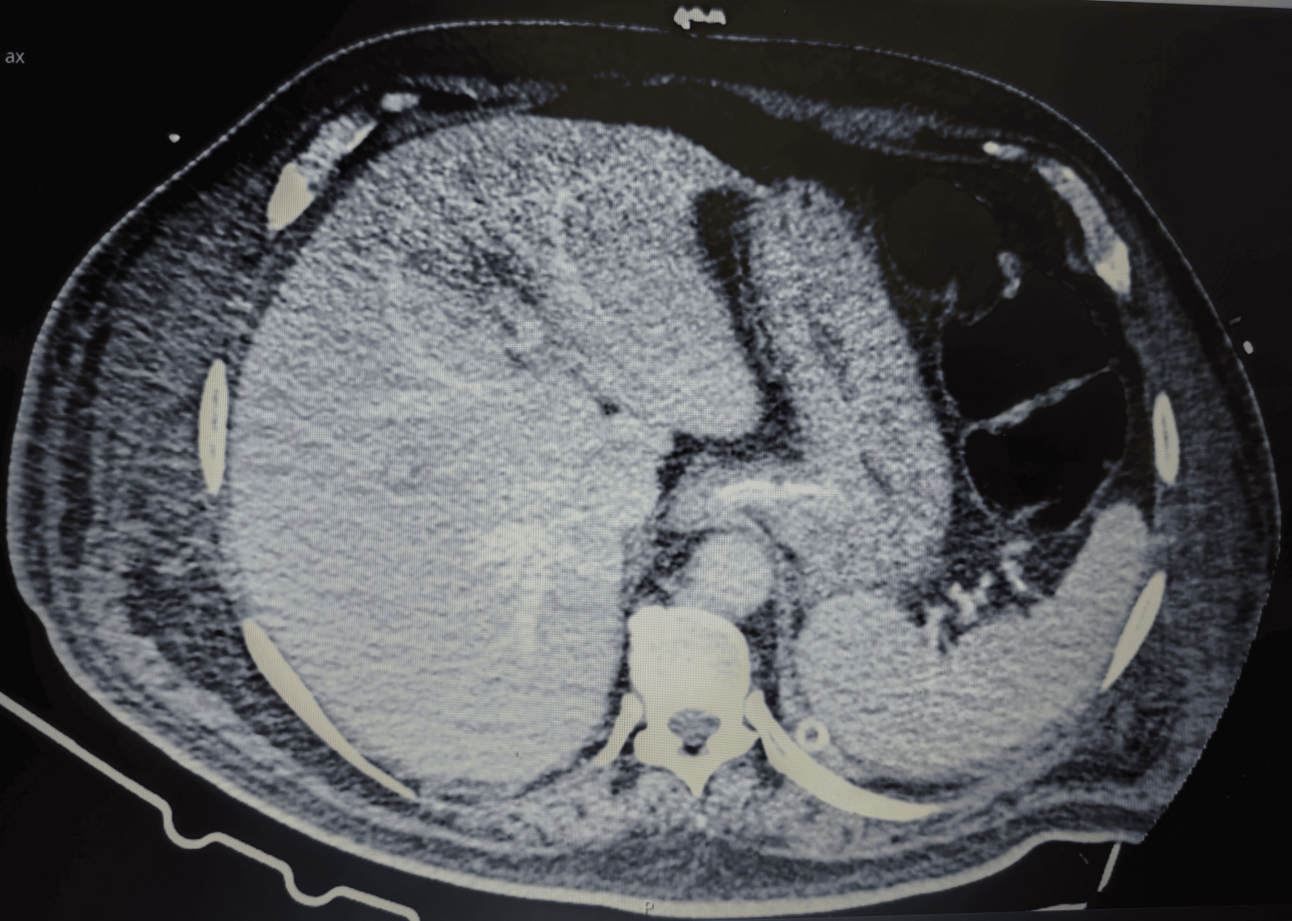
Abdominal computed tomography scan - portal venous phase

**Figure 2:**
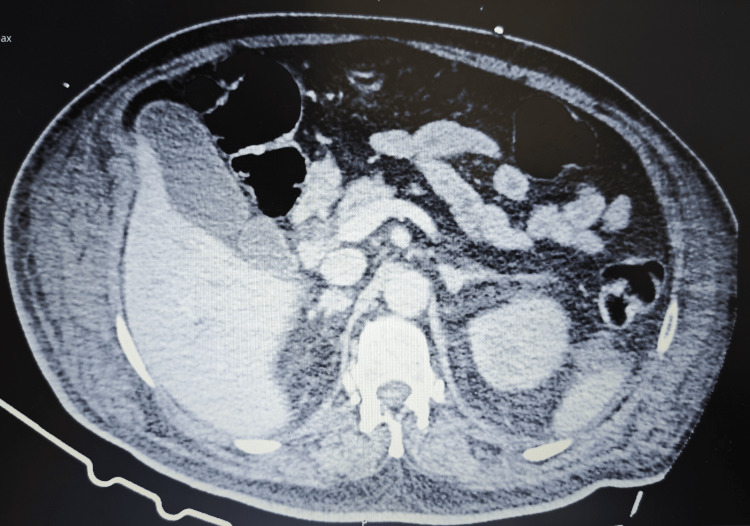
Abdominal computed tomography scan - portal venous phase

Despite these findings, liver tests continued to worsen, and a contrast-enhanced abdominal CT scan performed on day 18 (day 11 of meropenem therapy) confirmed hepatomegaly (19 cm) with homogeneous enhancement, but again with no evidence of biliary obstruction or other abnormalities (Figures 1-2). Concurrently, the patient developed a pruritic, maculopapular exanthem on the trunk, thighs, and skin folds, which resolved progressively after discontinuation of meropenem.

Due to significant hypoalbuminemia (1.7 g/dL), the patient received intravenous human albumin (20% solution, 20 g every eight hours for three days). Around the same time, he experienced progressive renal function deterioration, and continuous venovenous hemodiafiltration (CVVHDF) was initiated on day 19 and maintained until day 24.

Meropenem was discontinued on day 20, after a total of 12 days of therapy, with resolution of the pulmonary infection. Nevertheless, cholestatic parameters and total bilirubin continued to rise, despite drug withdrawal. A repeat abdominal ultrasound on day 26 again excluded any obstructive etiology.

On day 29, the patient developed a new infectious episode with ESBL-producing Proteus mirabilis bacteremia, for which ciprofloxacin was initiated. However, by day 30, the patient exhibited clinical signs of hepatic encephalopathy (West Haven grade II) and biochemical evidence of worsening cholestasis, with total bilirubin reaching 17.8 mg/dL. Despite the absence of hepatotoxic medications, hyperammonemia, or coagulopathy, he showed signs of somnolence, disorientation, and clonus. Given the worsening liver profile and encephalopathy, CVVHDF with hemoadsorption (HA-380 cartridge) was initiated.

Viral hepatitis screening (hepatitis A virus (HAV), hepatitis B virus (HBV), hepatitis C virus (HCV), hepatitis E virus (HEV); herpes simplex virus types 1 and 2 (HSV-1/2); varicella-zoster virus (VZV); Epstein-Barr virus (EBV); cytomegalovirus (CMV); human immunodeficiency virus types 1 and 2 (HIV-1/2), autoimmune markers, alpha-1-antitrypsin levels, serum ceruloplasmin and immunoglobulins were normal - ferritin was high but in an infectious/inflammatory context. 

Hemoadsorption therapy was maintained for four days, during which clinical and biochemical improvement was observed. However, upon cessation of therapy, liver enzymes and bilirubin levels again began to rise progressively.

On day 41, a liver biopsy was performed. Histopathological analysis revealed expansion of portal tracts with a mixed inflammatory infiltrate predominantly composed of neutrophils, occasional bile duct infiltration with degenerative changes, and a marked ductular reaction (CK7 positive) with ductular cholestasis. There was no significant interface activity. In the hepatic lobules, marked bilirubinostasis, scattered acidophil bodies, and a bile lake associated with CD163-positive macrophages were noted. No features of chronic cholestasis or iron/hyaline deposits were seen. These findings were consistent with intrahepatic cholestatic hepatitis with mild fibrosis. Given the clinical context and exclusion of other causes, DILI secondary to meropenem was deemed the most probable etiology, aligning with the morphologic patterns described in LiverTox (category D).

On day 44, with total bilirubin surpassing 20 mg/dL and the onset of severe pruritus, the patient resumed CVVHDF with hemoadsorption (HA-380 cartridge) and was started on ursodeoxycholic acid and hydroxyzine. A progressive reduction in liver enzymes and bilirubin was noted, with complete resolution of pruritus.

**Figure 3:**
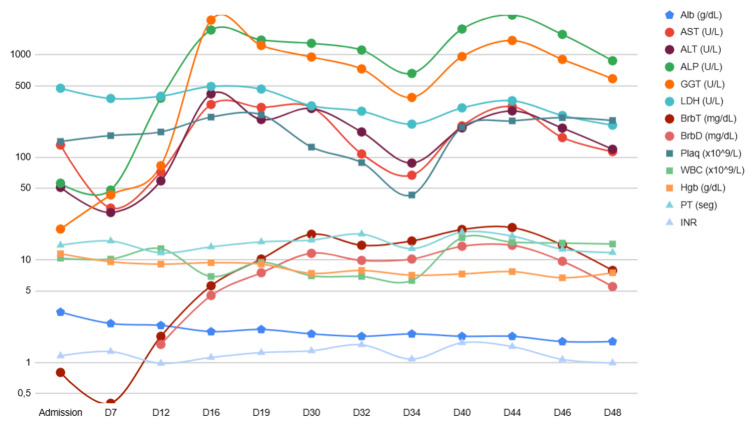
Laboratory results ALP: alkaline phosphatase; ALT: alanine aminotransferase; Alb: albumin; AST: aspartate aminotransferase; BrbD: direct bilirubin; BrbT: total bilirubin; GGT: gamma-glutamyl transferase; Hgb: hemoglobin; INR: international normalized ratio; LDH: lactate dehydrogenase; Plaq: platelets; PT: prothrombin time; WBC: white blood cells

The laboratory results and their evolution are shown in Table 1 and illustrated in Figure 3.

**Table 1:** Laboratory results ALP: alkaline phosphatase; ALT: alanine aminotransferase; AST: aspartate aminotransferase; BrbD: direct bilirubin; BrbT: total bilirubin; GGT: gamma-glutamyl transferase; Hgb: hemoglobin; INR: international normalized ratio; LDH: lactate dehydrogenase; Plaq: platelets; PT: prothrombin time; WBC: white blood cells

Day	Alb (g/dL)	AST (U/L)	ALT (U/L)	ALP (U/L)	GGT (U/L)	LDH (U/L)	BrbT (mg/dL)	BrbD (mg/dL)	Plaq ( ×10^9^/L)	WBC ( ×10^9^/L)	Hgb (g/dL)	PT (seg)	INR
D0	3.1	132	51	56	20	472	0.8	-	143	10.4	11.5	13.9	1.16
D7	2.4	32	29	48	43	375	0.4	-	163	10.2	9.6	15.3	1.28
D12	2.3	72	59	378	83	395	1.8	1.5	177	12.9	9.1	11.7	0.98
D16	2.0	328	416	1747	2181	492	5.6	4.5	247	6.9	9.4	13.4	1.12
D19	2.1	307	234	1393	1234	464	10.2	7.5	260	9.5	9.1	15.0	1.25
D30	1.9	308	300	1295	952	318	17.8	11.6	126	7.0	7.4	15.6	1.30
D32	1.8	108	177	1114	729	282	13.9	9.9	89	6.9	7.9	17.9	1.49
D34	1.9	67	88	658	384	211	15.3	10.2	43	6.3	7.1	12.9	1.08
D40	1.8	203	194	1785	962	304	19.8	13.6	197	16.5	7.3	18.7	1.56
D44	1.8	313	284	2440	1381	357	20.7	13.9	227	14.9	7.7	17.1	1.43
D46	1.6	156	193	1581	902	256	14.1	9.7	244	14.6	6.7	12.7	1.07
D48	1.6	114	120	875	585	206	7.9	5.5	229	14.3	7.5	11.8	0.99

The patient was subsequently transferred from the ICU to an intermediate care unit for ongoing rehabilitation and follow-up.

## Discussion

DILI remains a diagnostic challenge, particularly in critically ill patients who are exposed to multiple potentially hepatotoxic agents [2,3]. Meropenem, while generally considered safe, has been rarely associated with cholestatic hepatitis, often with delayed onset and a self-limited course [5-7]. In this case, a critically ill patient developed progressive hyperbilirubinemia and cholestatic liver enzyme elevation five days after initiating meropenem, in the absence of biliary obstruction or other identifiable causes. We can consider the patient's septic state as an aggravating factor.

The diagnosis of DILI is one of exclusion and was supported in this case by the temporal relationship between drug exposure and liver injury, histological confirmation of intrahepatic cholestatic hepatitis with features previously described in meropenem-associated injury, and the absence of other etiologies (e.g., viral, autoimmune, or obstructive) [1-3].

The role of hemoadsorption in the management of cholestatic DILI is not well established. However, in this case, the use of the HA-380 cartridge was temporally associated with significant reductions in bilirubin levels and symptomatic improvement in pruritus. While causality cannot be definitively established, the recurrence of cholestasis upon cessation of hemoadsorption and subsequent improvement after its reintroduction provides a compelling rationale for its therapeutic potential. Hemoadsorption has been proposed as a supportive therapy in various liver dysfunction contexts, particularly when bilirubin and cytokine clearance are desired, although current evidence remains limited to case reports and small series [8-10].

This case adds to the growing recognition of rare but severe hepatic reactions associated with meropenem and underscores the need for vigilance in monitoring liver function during prolonged antibiotic therapy, particularly in the ICU setting. We highlight the role of liver biopsy in the DILI study. It also suggests a potential role for extracorporeal therapies such as hemoadsorption in select cases of DILI, either as treatment or as a bridge to possible liver transplantation in irreversible cases, though controlled studies are needed.

## Conclusions

Meropenem-induced cholestatic liver injury, though rare, can present with significant biochemical abnormalities and clinical impact, especially in critically ill patients. This case illustrates the importance of early recognition of DILI and consideration of meropenem as a potential causative agent.

Early recognition and withdrawal of the offending agent, coupled with supportive therapies, such as hemoadsorption, may improve outcomes and prevent progression to irreversible liver damage. Hemoadsorption may be a valuable adjunctive therapy in severe cases with persistent hyperbilirubinemia and pruritus.
